# Defense Responses Induced by Viral Movement Protein and Its Nuclear Localization Modulate Virus Cell-to-Cell Transport

**DOI:** 10.3390/plants13182550

**Published:** 2024-09-11

**Authors:** Anastasia K. Atabekova, Ekaterina A. Lazareva, Alexander A. Lezzhov, Sergei A. Golyshev, Boris I. Skulachev, Sergey Y. Morozov, Andrey G. Solovyev

**Affiliations:** A. N. Belozersky Institute of Physico-Chemical Biology, Moscow State University, 119992 Moscow, Russia; asya_atabekova@mail.ru (A.K.A.); lazareva-katrina@mail.ru (E.A.L.); lezzhov-genetic@mail.ru (A.A.L.); sergei.a.golyshev@gmail.com (S.A.G.); bskulachev@gmail.com (B.I.S.); morozov@belozersky.msu.ru (S.Y.M.)

**Keywords:** plant virus, plant defense response, hypersensitive response, programmed cell death, movement protein, nuclear localization, nucleolus, Cajal bodies

## Abstract

Movement proteins (MPs) encoded by plant viruses are essential for cell-to-cell transport of viral genomes through plasmodesmata. The genome of hibiscus green spot virus contains a module of two MP genes termed ‘binary movement block’ (BMB), encoding the proteins BMB1 and BMB2. Here, BMB1 is shown to induce a defense response in *Nicotiana benthamiana* plants that inhibits BMB-dependent virus transport. This response is characterized by the accumulation of reactive oxygen species, callose deposition in the cell wall, and upregulation of 9-LOX expression. However, the BMB1-induced response is inhibited by coexpression with BMB2. Furthermore, BMB1 is found to localize to subnuclear structures, in particular to Cajal bodies, in addition to the cytoplasm. As shown in experiments with a BMB1 mutant, the localization of BMB1 to nuclear substructures enhances BMB-dependent virus transport. Thus, the virus transport mediated by BMB proteins is modulated by (i) a BMB1-induced defense response that inhibits transport, (ii) suppression of the BMB1-induced response by BMB2, and (iii) the nuclear localization of BMB1 that promotes virus transport. Collectively, the data presented demonstrate multiple levels of interactions between viral pathogens and their plant hosts during virus cell-to-cell transport.

## 1. Introduction

Plant virus genomes encode movement proteins (MPs), whose primary function is the cell-to-cell transport of viral genomic nucleic acid from infected cells to adjacent healthy cells through the plasmodesmata (PD) channels [1,2,3]. In addition, MPs can be involved in other functional interactions with host plants, serving as pathogenicity determinants, suppressors of RNA silencing, and/or avirulence factors [4,5,6]. The latter type of interaction with a plant host involves innate immunity, based on the plant resistance genes encoding proteins that specifically recognize, either directly or indirectly, viral polypeptides (avirulence factors) and induce a plant defense, often manifested as a hypersensitive response (HR), a form of programmed cell death (PCD) [7,8,9]. It is well documented that the MP of tomato mosaic virus (ToMV, genus *Tobamovirus*) is recognized as an avirulence factor in tomato plants carrying the resistance gene Tm-2^2^ [4,10]. The Tm-2^2^-mediated response to ToMV leads to so-called extreme resistance when the plant leaves exhibit no visible lesions after virus inoculation due to the blockage of viral infection at the level of primary infected cells [11]. Interestingly, the Tm-2^2^-dependent resistance is not observed upon virus infection of protoplasts; therefore, it has been hypothesized that the Tm-2^2^-mediated response operates at the level of virus cell-to-cell transport [12]. Indeed, the Tm-2^2^-encoded protein is found to function in the plasma membrane [13]. Another well-studied example of plant virus MPs, which can be specifically recognized in plants to induce HR, is the TGB1 protein of potato virus X (PVX, genus *Potexvirus*) [14]. The TGB1 protein contains an NTPase/helicase domain and is encoded together with two other MPs, TGB2 and TGB3, by the triple gene block (TGB), a transport gene module found in the genomes of a wide range of plant viruses [14]. In PVX-infected model plants, no HR can be observed; however, when the TGB1 expression level is elevated due to a silencing suppressor protein gene inserted into the PVX genome, systemic necrosis is observed in infected plants [5]. Similar effects are found upon coinfection of PVX and plum pox virus (PPV, genus *Potyvirus*) encoding the strong silencing suppressor Hc-Pro, or in plants infected with PPV expressing the artificially introduced PVX TGB1 gene. According to the suggested concept, HR can be triggered by TGB1 once its amount in plant cells reaches a threshold level [5]. In a similar manner, the strength of plant defense response mediated by the Tm-2^2^ gene depends on its expression level and can vary from (1) extreme resistance that blocks virus spread from primary infected cells to (2) HR, allowing limited local virus transport out of the primary cells and subsequent necrosis of the local infected area; and (3) a weaker response that cannot block systemic virus transport and is manifested by systemic necrosis [13]. Therefore, based on studies of PVX TGB1 and the ToMV/Tm-2^2^ system, it can be concluded that the development of a plant defense response depends on relative amounts of the viral avirulence factor and cognate resistance gene.

To mediate virus cell-to-cell transport, MPs operate in the cytoplasm and PD. However, some MPs of positive-stranded RNA viruses, whose genomes replicate in the cytoplasm, have been found in the nucleus. Among these proteins, the most studied is the TGB1 protein. In virus-infected cells, the TGB1 protein of potato mop-top virus (PMTV, genus *Pomovirus*) is found in PD and in the cytoplasm in association with microtubules, as well as in the nucleus and nucleolus [15]. As revealed by mutagenesis, the N-terminal region of PMTV TGB1 contains two nucleolar localization signals (NoLS), which are essential for both the protein localization to the nucleolus and virus systemic transport [16]. The TGB1 protein of poa semilatent virus (PSLV, genus *Hordeivirus*), which is mainly localized in the cytoplasm, is also found in the nucleolus, probably due to the nuclear localization signal (NLS) and NoLS mapped to the N-terminal region of the protein, and interacts with the major nucleolar protein fibrillarin (Fib2) [17]. In addition, the PSLV TGB1 interacts with coilin, a protein characteristic of Cajal bodies, another type of subnuclear structures [18]. Similarly, TGB1 of another hordeivirus, barley stripe mosaic virus (BSMV), localizes to the nucleolus and has NLS and NoLS, which are both of functional significance, as the NoLS and NLS mutations suppress and almost completely inhibit virus cell-to-cell transport, respectively, and fully block the virus systemic spread [19]. BSMV TGB1 interacts with Fib2, and the BSMV transport in *fib2* knockdown plants is inhibited [19]. Conceivably, the function of Fib2 binding by hordeivirus TGB1 proteins can be similar to that of Fib2 binding by the ORF3 protein of groundnut rosette virus (GRV, genus *Umbravirus*), the protein not involved in virus cell-to-cell movement but required for systemic transport [20]. The GRV ORF3 protein interacts with Fib2 in the nucleus and induces Fib2 export to the cytoplasm, where it takes part, together with the ORF3 protein, in the formation of complexes with GRV genomic RNA that are capable of systemic transport through the phloem [21,22]. Therefore, the hordeivirus TGB1 can be speculated to recruit Fib2 for the formation of similar complexes destined for cell-to-cell transport. There are a few other known, although less studied, nucleus-localized MPs. One example of such proteins is the turnip vein-clearing virus (TVCV, genus *Tobamovirus*) MP. It partly localizes to the nucleus, being found in filaments associated with chromatin, and an NLS found in TVCV MP is necessary for efficient virus cell-to-cell transport [23]. Nuclear localization is also shown for p8, one of the two MPs encoded by turnip crinkle virus (genus *Betacarmovirus*); however, mutations introduced in two NLSs found in p8, although they suppress protein nuclear targeting, have no effect on the efficiency of virus movement [24], leaving the functional significance of p8 nuclear localization unclear.

Binary movement block (BMB) is a transport module first described in the genome of hibiscus green spot virus (HGSV, genus *Higrevirus*), consisting of two genes encoding MPs called BMB1 and BMB2 [25,26]. BMB1 is distantly related to the TGB1 proteins and contains an NTPase/helicase domain [27,28]. BMB1 is known to localize to both the cytoplasm and the nucleoplasm [26]. The functions of HGSV BMB1 in virus movement have not been studied; however, by analogy to TGB1 proteins, BMB1 is assumed to bind viral genomic RNA to form complexes that are competent for cell-to-cell transport. BMB2 is a small hydrophobic protein that is integrated into the membranes of the endoplasmic reticulum (ER); by adopting a W-like topology in the membrane and forming high molecular weight complexes, BMB2 acts like reticulons, cell proteins that generate lipid bilayer curvature [29]. Upon transient expression in plant cells, BMB2 induces local constrictions of the ER tubules and the formation of ER-derived membrane compartments located in close vicinity of PD, termed PD-associated membrane bodies (PAMBs) [29,30]. As shown by electron tomography, PAMBs are formed by membrane cisterns derived from and connected to the ER tubules; the cisterns are linked by numerous intermembrane contact sites that likely hold the PAMB structure together [31]. Upon coexpression with BMB1, BMB2 directs BMB1 to PAMBs due to the interaction between BMB1 and BMB2 molecules [32], to the PD interior, and to neighboring cells through the PD channels [26]. The latter BMB2 function can be explained by the BMB2 ability to modify PD and increase their conductivity [29]. Therefore, according to the current model of BMB functioning, BMB1 likely binds viral genomic RNA, while BMB2 modifies the ER membranes to deliver the BMB1-containing complexes to PD and further through PD to adjoining cells. Recently, deletion of the BMB1 22 C-terminal amino acid residues has been found to considerably enhance the BMB1 association with subnuclear structures that are barely detectable for the wild-type protein [32], suggesting that BMB1 can have additional, nuclear-specific functions.

In this paper, BMB1 is shown to be an inducer of plant defense responses, which are suppressed under the conditions of coexpression with BMB2. It is also shown that BMB1 localizes to Cajal bodies and that the nuclear localization of BMB1 promotes virus cell-to-cell transport.

## 2. Results

### 2.1. BMB1 Expression Induces Defense Response in N. benthamiana

**Figure 1:**
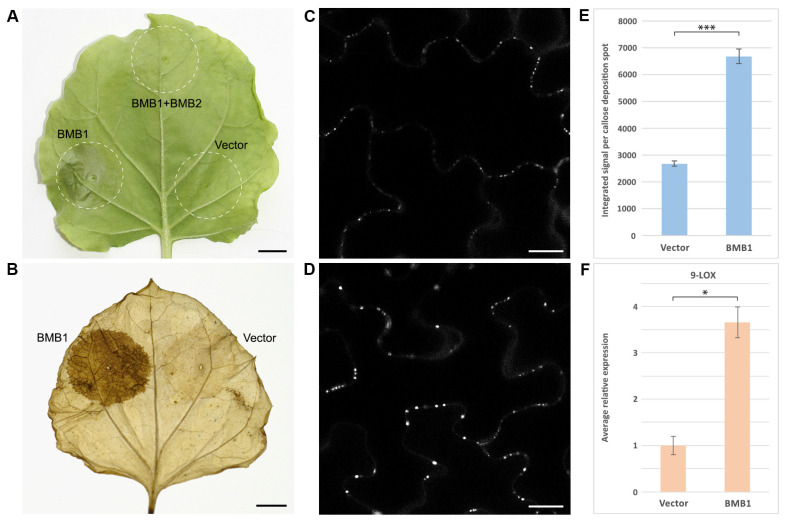
BMB1 induces a defense response in *N. benthamiana*. (**A**) A PCD-like defense response in a leaf region agroinfiltrated for expression of BMB1 and coexpression of BMB1 and BMB2. An empty vector was used as a control. The leaf was imaged at 5 dpi. (**B**) ROS accumulation in leaves agroinfiltrated for expression of BMB1. DAB staining was carried out at 3 dpi. Scale bars in (**A**,**B**), 1 cm. (**C**,**D**) Staining of callose depositions with aniline blue in cell walls of leaves agroinfiltrated for expression of empty vector (**C**) and BMB1 (**D**) at 2 dpi. Typical images are shown. Scale bar in (**C**,**D**), 20 μm. (**E**) Quantification of callose staining data. Average integrated intensities of signal calculated for individual callose deposition spots are shown; error bars indicate the standard error. More than 500 individual callose deposition spots were measured on five agroinfiltrated leaves to calculate the values shown. Asterisks indicate a statistically significant difference (***, *p* < 0.001) according to the Student’s *t*-test. (**F**) The expression level of 9-LOX in leaves agroinfiltrated for expression of empty vector and BMB1. Samples were collected at 2 dpi. Average expression levels determined by qPCR are shown; error bars indicate the standard error. Ten biological replicates were used to calculate each value shown. The asterisk indicates a statistically significant difference (*, *p* < 0.5) according to the Student’s *t*-test.

*Nicotiana benthamiana* leaves agroinfiltrated for expression of HGSV BMB1 exhibited senescence, dying, and necrotization of leaf tissue in the infiltrated areas at 4 to 5 days post infiltration (dpi) (Figure 1A), thus showing the plant tissue reaction resembling the HR. No such response was observed at these time points in the control leaf areas infiltrated with the agrobacterial culture carrying an empty expression vector (Figure 1A). To verify that the BMB1-induced HR-like response is not only induced under the conditions of agrobacteria-mediated expression, another method of BMB1 delivery to plant cells was used. To this end, the BMB1 gene was cloned in a tobacco rattle virus (TRV) expression vector [33]. In contrast to control plants infected with an empty vector, TRV-BMB1-infected plants exhibited an HR-like reaction in inoculated and systemically infected leaves, often manifested as leaf tissue death at sites of virus unloading from the phloem in upper leaves (Supplementary Figure S1), confirming that the BMB1-induced defense response does not depend on the method of BMB1 expression.

To characterize the response to BMB1 expression, leaves agroinfiltrated for BMB1 expression were stained with 3,3′-diaminobenzidine (DAB), which is often used to detect reactive oxygen species (ROS) in histochemical reactions, as it is oxidized by hydrogen peroxide to give a color derivative. The DAB staining revealed that leaf areas agroinfiltrated for BMB1 expression, but not those agroinfiltrated with an empty vector, exhibited brown coloring that is typical of oxidized DAB (Figure 1B). Plant defense responses often involve callose deposition in the cell wall [34]; therefore, leaves agroinfiltrated for BMB1 expression were stained with aniline blue, a fluorescent dye that specifically stains callose. As shown by confocal laser scanning microscopy, the callose-specific fluorescent signal in the cell walls was significantly brighter in the BMB1-expressing leaf areas than in the empty vector-agroinfiltrated control areas (Figure 1C,D). Quantification of the callose-specific signal revealed that callose deposition in the cell wall increased 2.5-fold upon BMB1 expression (Figure 1E). Since genes of the oxylipin biosynthesis pathway are known to positively regulate PCD induced by virus infection and are upregulated during the development of virus-induced PCD [35], the expression level of 9-lipoxygenase (9-LOX) was measured in leaf areas agroinfiltrated for BMB1 expression. According to the qPCR data, BMB1 expression resulted in a 3.7-fold increase in the 9-LOX mRNA level compared to the control (Figure 1F). Therefore, expression of BMB1 in *N. benthamiana* leaves induced a defense response accompanied by ROS accumulation, callose deposition in the cell wall, and upregulation of 9-LOX expression.

### 2.2. BMB2 Suppresses Defense Response Induced by BMB1

**Figure 2:**
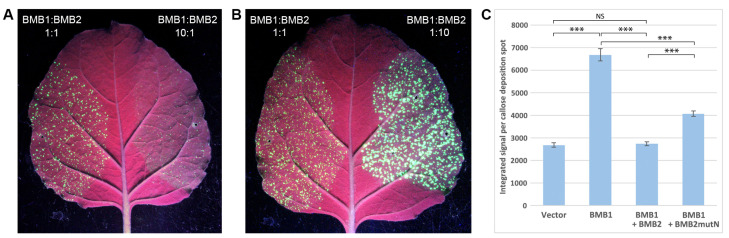
BMB2 suppresses the defense response induced by BMB1. (**A**,**B**) The efficiency of virus transport depends on the ratio of BMB1 to BMB2. In the virus transport complementation assay, *N. benthamiana* leaves were agroinfiltrated for coexpression of PVX-POL-GFP with a combination of BMB1 and BMB2 at a BMB1:BMB2 ratio of either 1:1 ((**A**,**B**), left leaf halves), 10:1 ((**A**), right leaf half), or 1:10 ((**B**), right leaf half). Leaves were imaged under UV light at 4 dpi. (**C**) BMB2 suppresses callose deposition induced by BMB1 expression, as determined by quantification of callose staining data obtained for leaves agroinfiltrated for expression of vector, BMB1, BMb1 + BMB2, or BMB1 + BMB2mutN. Average integrated signal intensities calculated for individual callose deposition spots are shown; error bars indicate the standard error. More than 500 individual callose deposition spots were measured on five agroinfiltrated leaves to calculate the values shown. Asterisks indicate a statistically significant difference (***, *p* < 0.001) according to Student’s *t*-test. NS, not statistically significant (*p* > 0.05).

In previous experiments, to analyze the BMB1/BMB2 movement function, a complementation test using PVX-POL-GFP, a reporter construct derived from the PVX genome [36], was used. In PVX-POL-GFP, the viral genome region, including TGB and the capsid protein (CP) gene, has been replaced by a GFP gene under the control of the CP subgenomic promoter, which directs GFP expression in infected cells [36]. Therefore, PVX-POL-GFP is capable of replication in initially infected cells but is incapable of cell-to-cell transport; however, this ability can be restored by coexpression with BMB1 and BMB2, resulting in the formation of multicellular infection loci that are readily detectable under UV light due to GFP expression [36]. In routine PVX-POL-GFP transport complementation experiments, a BMB1:BMB2 ratio of 1:1 is used [26,32,36]. However, we found that the efficiency of virus transport, as manifested by the size of fluorescent loci, appeared to be highly dependent on the BMB1:BMB2 ratio. When agrobacterial cultures for expression of BMB1 and BMB2 were mixed at a BMB1:BMB2 ratio of 10:1 prior to infiltration of *N. benthamiana* leaves, the size of the fluorescent loci was significantly reduced compared to those at a BMB1:BMB2 ratio of 1:1 (Figure 2A). In contrast, when a BMB1:BMB2 ratio of 1:10 was used, the size of the loci was larger compared to a BMB1:BMB2 ratio of 1:1 (Figure 2B). We hypothesized that the suppressed virus transport observed at high BMB1 expression levels could be explained by a stronger BMB1-induced defense response that limited virus cell-to-cell transport. On the other hand, the enhanced virus transport observed at high BMB2 expression levels could be due to the BMB2 ability to increase the PD conductivity [29], which, at high levels of BMB2 accumulation, would result in more processive virus transport. Alternatively, the observed BMB2 effect could be due to the ability of BMB2 to suppress the BMB1-induced plant defense response.

To determine whether BMB2 can suppress the defense response induced by BMB1, *N. benthamiana* leaves were agroinfiltrated for coexpression of BMB1 and BMB2. At 5 dpi, when expression of BMB1 resulted in senescence and necrotization of leaf tissues, no such reaction was observed in the leaf area infiltrated for coexpression of BMB1 and BMB2 (Figure 1A). To analyze whether this phenotypic effect is accompanied by the suppression of the plant defense response, the level of callose accumulation in the cell wall was measured in leaf areas coexpressing BMB1 and BMB2 in comparison with areas where BMB1 was coexpressed with an empty vector. Quantification of the callose staining data showed that the level of callose accumulation was considerably reduced upon coexpression with BMB2 and did not show a statistically significant difference from that in leaves agroinfiltrated for empty vector expression (Figure 2C), demonstrating the ability of BMB2 to suppress the BMB1-induced plant response. To determine whether this suppression could result from the BMB2-directed targeting of BMB1 to PAMBs, the previously described BMB2 mutant BMB2mutN [32] was used. This mutant contains amino acid substitutions in the N-terminal region of BMB2 and is deficient in its interaction with BMB1 [32]. Quantification of callose analysis data for coexpression of BMB1 and BMB2mutN revealed that callose deposition was higher compared to coexpression of BMB1 and BMB2 and lower compared to coexpression of BMB1 with empty vector (Figure 2C). These data show that the interaction between BMB1 and BMB2 contributes to the suppression of the BMB1-induced plant defense response by BMB2, and indicate that other factors besides this interaction may be involved in the BMB2-dependent suppression of the BMB1-induced response.

### 2.3. BMB1 Is Partially Localized to Nuclear Substructures

**Figure 3:**
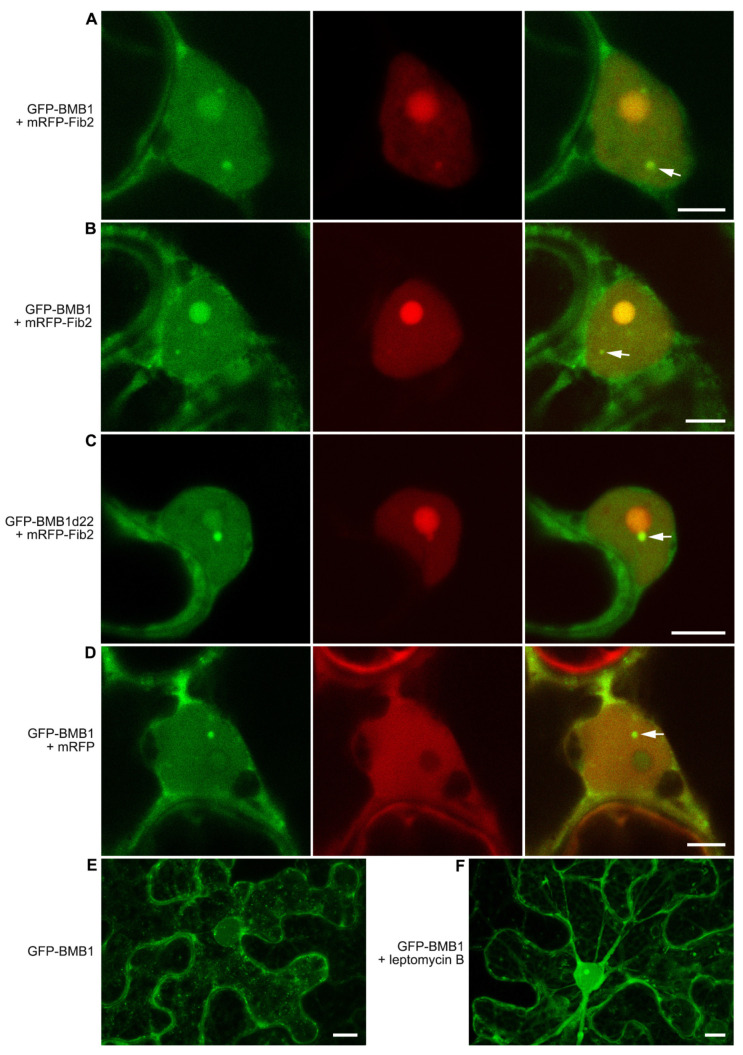
Localization of BMB1 to nuclear substructures. (**A**,**B**) Coexpression of GFP-BMB1 with mRFP-Fib2. In different individual cells, moderate (**A**) or high (**B**) levels of GFP-BMB1 accumulation in the nucleolus were observed upon coexpression with mRFP-Fib2. (**C**) Coexpression of GFP-BMB1d22 with mRFP-Fib2. (**D**) Coexpression of GFP-BMB1 with mRFP. (**E**,**F**) Treatment with leptomycin B results in the accumulation of GFP-BMB1 in the nucleus (**F**) compared to an untreated cell expressing GFP-BMB1 (**E**). In (**A**–**D**), the left images represent the GFP channel, the center images represent the mRFP channel, and the right images are a superposition of the images for the GFP and mRFP channels. Arrows point to Cajal bodies. Scale bars: 5 μm in (**A**–**D**,**F**); 10 μm in (**E**,**F**).

Previously, BMB1 has been detected, in addition to the cytoplasm and nucleoplasm, in a small subnuclear body, and localization to this body was significantly enhanced in a BMB1 mutant with 22 C-terminal amino acid residues deleted (BMB1d22) [32]. As this subnuclear structure was reminiscent of Cajal bodies [37], *Arabidopsis thaliana* fibrillarin-2 fused to mRFP (mRFP-Fib2), a marker known to localize to the nucleolus and Cajal bodies, was used to identify these structures. Therefore, *N. benthamiana* leaves were agroinfiltrated for coexpression of mRFP-Fib2 with either GFP-BMB1 or GFP-BMB1d22. As determined by confocal microscopy at 3 dpi, the BMB1-specific subnuclear bodies colocalized with mRFP-Fib2-labeled Cajal bodies in both GFP-BMB1- and GFP-BMB1d22-expressing cells (Figure 3A–C).

It should be noted that coexpression with mRFP-Fib2 resulted in a concentration of BMB1 in the nucleolus, which was manifested to varying degrees in the cells examined (Figure 3A,B) and was never observed in the control coexpression of GFP-BMB1 with mRFP (Figure 3D). Interestingly, in many cells expressing GFP-BMB1, the intensity of the GFP fluorescence was similar in the nucleoplasm and the nucleolus and was visibly lower at the boundary of these two compartments, forming a characteristic low-fluorescence ring surrounding the nucleolus (Figure 3D). Collectively, these data indicate that BMB1 has an affinity for Cajal bodies and that this affinity increased upon deletion of the BMB1 C-terminal region.

In additional experiments, BMB1-expressing cells were analyzed to determine whether BMB1 localization in the nucleus and Cajal bodies was static or dynamic. Samples of leaves agroinfiltrated for GFP-BMB1 expression were treated with leptomycin B, a compound known to inhibit CRM1-dependent nuclear export of proteins [38]. As shown by confocal microscopy, leptomycin B treatment resulted in an increased amount of GFP-BMB1 in the nucleus compared to untreated cells (Figure 3E). These data indicate that BMB1 is not only capable of nuclear import but also of nuclear export, suggesting a nuclear-cytoplasmic shuttling of BMB1.

### 2.4. Search for Signals Directing BMB1 to Nuclear Substructures

Sequence analysis of the BMB1 protein revealed the absence of obvious nuclear/nucleolar localization signals, which were therefore attempted to be identified by mutagenesis. The search for possible targets for mutagenesis was carried out using services that predict regions located on the surface of a protein molecule, including NetSurfP, PredictProtein, WESA, DeepREx-WS, and SVMTriP (see Materials and Methods). As a result, four internal regions of BMB1 corresponding to residues 97–102, 111–115, 238–243, and 287–291 were selected, as they were confidently predicted to be surface-located. Site-directed mutagenesis was used to make two point substitutions in each of these regions. As a result, the following four BMB1 mutants were obtained: BMB1-mut1 (with mutations K98S, R99G), BMB1-mut2 (S112A, R114S), BMB1-mut3 (K238G, Y240S), and BMB1-mut4 (R287G, K291G). The subcellular localization of the GFP-fused mutants was examined in agroinfiltrated *N. benthamiana* leaves. All mutants were found to retain the localization characteristic of the wt BMB1 protein (Supplementary Figure S2). Thus, none of the introduced mutations blocked protein localization to the nucleus or shifted the dynamic ratio of protein distribution between the nucleus and the cytoplasm, leaving unidentified signals that direct the nuclear trafficking of BMB1.

### 2.5. Analysis of BMB1 with Artificially Added NES or NLS

**Figure 4:**
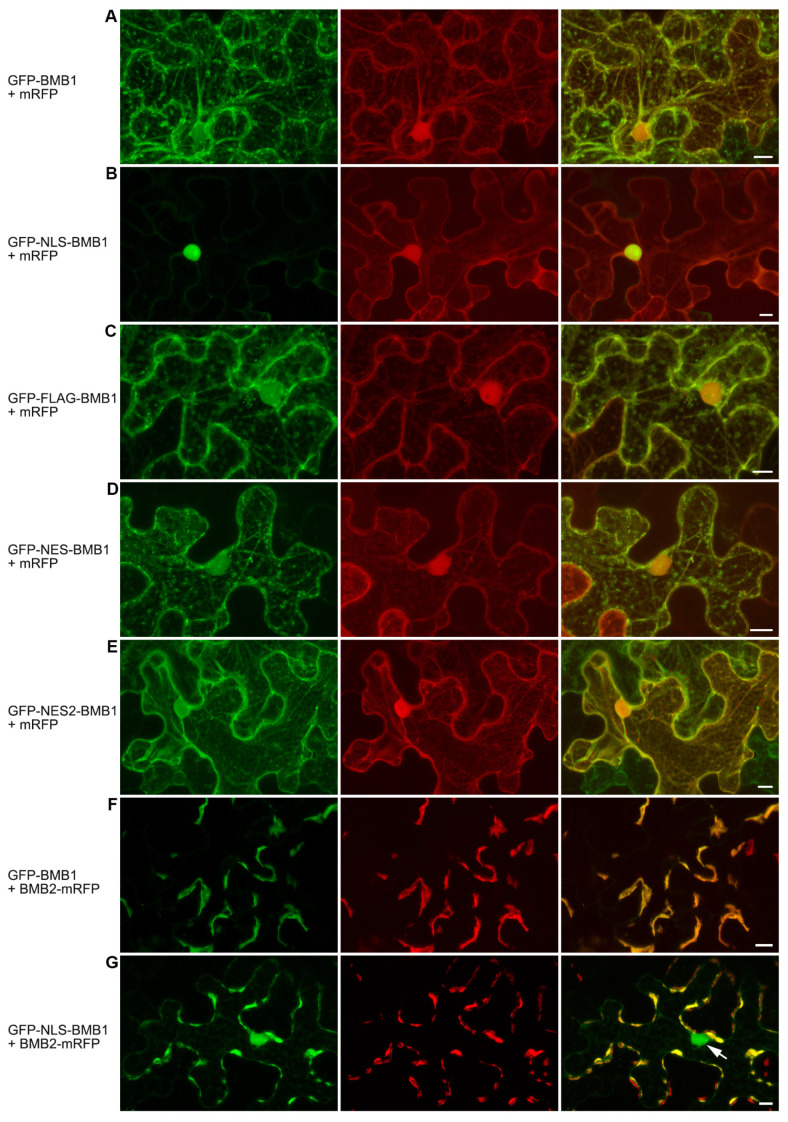
Subcellular localization of BMB1 with artificially added NLS, NES, or FLAG. Confocal images show cells of *N. benthamiana* leaves agroinfiltrated for coexpression of GFP-BMB1 and mRFP (**A**), GFP-NLS-BMB1 and mRFP (**B**), GFP-FLAG-BMB1 and mRFP (**C**), GFP-NES-BMB1 and mRFP (**D**), GFP-NES2-BMB1 and mRFP (**E**), GFP-BMB1 and BMB2-mRFP (**F**), and GFP-NLS- BMB1 and BMB2-mRFP (**G**). The arrow in (**G**) points to the nucleus. The left images represent the GFP channel, the center images represent the mRFP channel, and the right images are a superposition of the images for the GFP and mRFP channels. Scale bars: 10 μm.

As the natural BMB1 nuclear trafficking signals could not be identified, to analyze specific protein functions in the cytoplasm and nucleus, the BMB1 protein was modified to carry artificially added exogenous nuclear trafficking signals, namely the nuclear localization signal (NLS) of the SV40 T-antigen (PKKKRKVEDP) [39] and the nuclear export signal (NES) of the cAMP-dependent protein kinase inhibitor protein (ELALKLAGLDIN) [40]. A FLAG epitope (DYKDDDDK) [41] was used as a control. The NLS, NES, and FLAG sequences were added to the BMB1 N-terminus because, as previously shown, the addition of exogenous sequences such as GFP to the BMB1 N-terminus had no effect on the protein functions in cell-to-cell transport [26], while the protein C-terminus was involved in the interaction with BMB2 [32]. To analyze the subcellular localization of the obtained fusion proteins NLS-BMB1, NES-BMB1, and FLAG-BMB1, these constructs were modified to carry GFP at their N-termini (Supplementary Figure S3). Leaves of *N. benthamiana* plants were agroinfiltrated for expression of GFP-NLS-BMB1, GFP-NES-BMB1, and GFP-FLAG-BMB1, and the subcellular localization of the fusions was examined by confocal microscopy. GFP-BMB1 was used as a control. As expected, the addition of NLS to BMB1 led to the accumulation of the protein in the nucleus (Figure 4B), whereas the addition of FLAG did not affect the localization of the protein (Figure 4C). However, the addition of NES had no visible effect on protein localization (Figure 4D). Therefore, in a further attempt to obtain the cytoplasmically localized version of BMB1, the protein kinase inhibitor NES in the GFP-NES-BMB1 construct was replaced by the HIV-1 Rev NES (LQLPPLERLTL) [42] to give GFP-NES2-BMB1. The subcellular localization of this fusion protein appeared indistinguishable from that of GFP-BMB1 (Figure 4E). Thus, neither NES sequence added to BMB1 had the expected effect on protein localization. Therefore, only GFP-NLS-BMB1, which was apparently concentrated in the nucleus, was used in further experiments.

**Figure 5:**
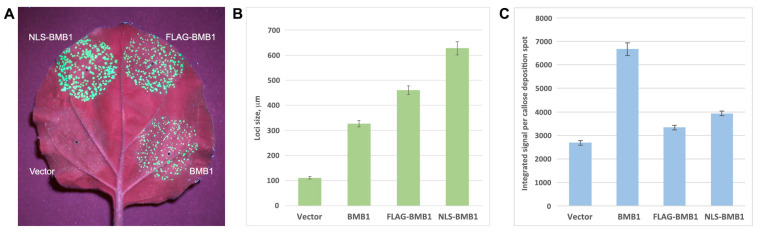
Influence of NLS and FLAG added to the N-terminus of BMB1 on protein functions. (**A**) Influence of NLS and FLAG on virus cell-to-cell transport in the complementation assay. *N. benthamiana* leaves were agroinfiltrated for coexpression of PVX-POL-GFP and BMB2 with either empty vector, BMB1, FLAG-BMB1, or NLS-BMB1 as indicated. The leaf was imaged under UV light at 4 dpi. (**B**) Quantification of the diameter of infection foci in the complementation experiment. At least 30 loci were measured on four agroinfiltrated leaves to calculate each average value. Error bars indicate the standard error. (**C**) Quantification of callose deposition staining data for leaf areas agroinfiltrated for expression of empty vector, BMB1, FLAG-BMB1, or NLS-BMB1. More than 500 individual callose deposition spots were measured on five agroinfiltrated leaves to calculate the plotted average values. Error bars indicate the standard error. Differences between all pairs of values plotted in (**B**,**C**) are statistically significant (*p* < 0.001).

As previously shown, GFP-BMB1 can be targeted by BMB2 to PAMBs containing BMB2 [26]. To analyze whether the BMB1 carrying NLS was capable of BMB2-dependent targeting to PAMBs, *N. benthamiana* leaves were agroinfiltrated for coexpression of GFP-NLS-BMB1 and BMB2-mRFP. In the control, when a combination of GFP-BMB1 and BMB2-mRFP was used, GFP fluorescence completely colocalized with BMB2-mRFP-containing PAMBs (Figure 4F), whereas GFP-NLS-BMB1 localized to both PAMBs and the nucleus (Figure 4G). These observations demonstrate that GFP-NLS-BMB1 retains the ability to be targeted by BMB2 to PAMBs. To analyze the functional competence of NLS-BMB1, a complementation assay using PVX-POL-GFP was carried out [36]. As shown by the observations under UV light, when *N. benthamiana* leaves were agroinfiltrated for PVX-POL-GFP expression with a highly diluted agrobacterial culture, GFP fluorescence was observed in single cells, whereas coexpression of PVX-POL-GFP with both BMB1 and BMB2 resulted in the formation of multicellular infection loci (Figure 5A) due to complementation of PVX-POL-GFP cell-to-cell transport as described previously [36]. Coexpression of PVX-POL-GFP with BMB2 and either NLS-BMB1 or FLAG-BMB1 also resulted in the formation of multicellular fluorescent loci, demonstrating that none of the added peptides abolished the transport functions of BMB1. Surprisingly, in the presence of BMB2, both NLS-BMB1 and FLAG-BMB1 induced the formation of loci of larger sizes than wt BMB1 protein, with the effect of NLS-BMB1 being more pronounced than that of FLAG-BMB1 (Figure 5A). Measurements of the size of the loci confirmed the visual observations and revealed statistically significant differences between the size of the loci formed in the presence of BMB1, NLS-BMB1, and FLAG-BMB1 (Figure 5B). Therefore, the addition of NLS and FLAG to the N-terminus of BMB1 results in more processive virus transport in the complementation assay.

The observation that the addition of two foreign peptides to the N-terminus of BMB1 enhanced virus cell-to-cell transport may indicate that these peptides inhibit the induction of the plant response to BMB1 expression. To verify this hypothesis, *N. benthamiana* leaves were agroinfiltrated for expression of BMB1, NLS-BMB1, and FLAG-BMB1, and an empty vector was used as a negative control. The defense response was evaluated by quantifying callose deposition in the cell wall. Both NLS and FLAG were found to significantly inhibit the defense response induced by BMB1 (Figure 5C). Importantly, the callose deposition was inhibited to a similar extent for NLS-BMB1 and FLAG-BMB1 (Figure 5C); however, the loci sizes in the case of NLS-BMB1 were larger than those observed for FLAG-BMB1 (Figure 5B). These observations may suggest that the nuclear localization of BMB1 due to the added artificial NLS is the cause of the enhanced virus transport observed for NLS-BMB1 compared to FLAG-BMB1. Taken together, these data show that NLS and FLAG added to the N-terminus of BMB1 suppress the BMB1-induced plant defense response, thereby enhancing virus transport; in addition, BMB1 localized to the nucleus can further enhance virus transport, probably due to its interaction with nuclear proteins.

### 2.6. Dysfunctional BMB1 Localized to the Nucleus Enhances Virus Transport

**Figure 6:**
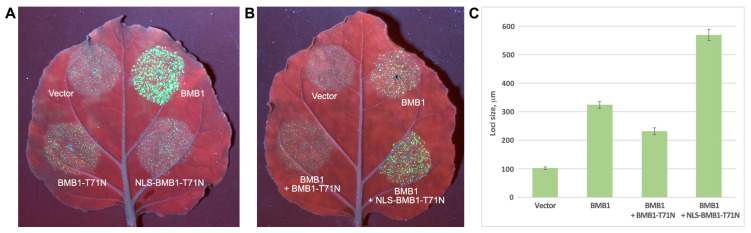
Dysfunctional BMB1 localized to the nucleus enhances virus transport. (**A**) Competence of BMB1-T71N and NLS-BMB1-T71N in virus transport. In a virus transport complementation assay, *N. benthamiana* leaves were agroinfiltrated for coexpression of PVX-POL-GFP with BMB2, and either empty vector, BMB1, BMB1-T71N, or NLS-BMB1-T71N. Leaves were imaged under UV light at 4 dpi. (**B**) Influence of dysfunctional BMB1 derivatives on virus cell-to-cell transport. In a virus transport complementation assay, *N. benthamiana* leaves were agroinfiltrated for coexpression of PVX-POL-GFP with BMB1, BMB2, and either BMB1-T71N or NLS-BMB1-T71N. Leaves were imaged under UV light at 4 dpi. (**C**) Quantification of the diameter of infection foci in the complementation experiment. At least 30 loci were measured on four agroinfiltrated leaves to calculate each average value. Error bars indicate the standard error. Differences between all pairs of values plotted in (**C**) are statistically significant (*p* < 0.001).

To directly test whether the nuclear localization of BMB1 can promote virus cell-to-cell transport, a BMB1 mutant was constructed by introducing a point mutation into the conserved motif I of the BMB1 NTPase/helicase domain to replace a Thr residue with an Asn residue at the position 71 of the protein (T71N). Such a substitution is known to block the enzymatic activity of NTPase domains and to inhibit viral transport in the case of TGB1 proteins [14]. The resulting construct BMB1-T71N was further modified by adding NLS to the N-terminus of the protein to give NLS-BMB1-T71N. As expected, in the presence of BMB2, neither BMB1-T71N nor NLS-BMB1-T71N could enable virus transport in the complementation assay (Figure 6A), confirming that the introduced mutation rendered the protein dysfunctional in mediating virus cell-to-cell transport. Analysis of the ability of the mutants to induce plant defense responses was carried out by DAB staining of *N. benthamiana* leaves agroinfiltrated for expression of BMB1-T71N or NLS-BMB1-T71N; wt BMB1 was used as a control. In these experiments, BMB1-T71N retained the ability to induce the defense response (Supplementary Figure S4), whereas, in the case of NLS-BMB1-T71N, the response was considerably suppressed (Supplementary Figure S4), which is consistent with a similar suppression observed for NLS-BMB1 (see above). To analyze the effect of dysfunctional BMB1 derivatives on virus transport, *N. benthamiana* leaves were infiltrated with an agrobacterial mixture for coexpression of BMB1 and BMB2 supplemented with an agrobacterial culture for expression of BMB1-T71N, NLS-BMB1-T71N, or an empty vector. Observation of the infiltrated leaves under UV light demonstrated that the addition of BMB1-T71N to the BMB1 + BMB2 combination resulted in the formation of infection loci of smaller sizes compared to BMB1 + BMB2 (Figure 6B). In contrast, the addition of NLS-BMB1-T71N resulted in enlarged virus infection loci (Figure 6B). Visual observations were confirmed by the measurements of the loci sizes that demonstrated statistically significant changes in loci size for both BMB1-T71N and NLS-BMB1-T71N (Figure 6C). These data suggest that the addition of BMB1-T71N, which is capable of inducing the defense response, enhanced the plant response that led to the restriction of virus transport. On the other hand, the observation that the addition of NLS-BMB1-T71N, which is dysfunctional in virus transport and unable to induce the defense response, enhances virus transport suggests that the nucleus-targeted BMB1 can indeed promote virus cell-to-cell transport independently of the enzymatic functions of BMB1 that are essential for virus transport.

## 3. Discussion

The HGSV transport system consists of two MPs, BMB1 and BMB2 [26]. In this paper, we demonstrate that BMB1 induces a plant defense response in *N. benthamiana*, while BMB2 is able to suppress the BMB1-induced response. The plant defense response is found to be induced upon transient expression of BMB1 by agroinfiltration and thus does not require viral infection, viral dsRNA, or other virus-specific products. Therefore, BMB1 can be considered as a viral elicitor of plant defense; however, the response induced by BMB1 is different from that observed in the case of incompatible plant–pathogen interaction, which is determined by the specific recognition of a pathogen avirulence factor in plants carrying the corresponding resistance gene [9]. For example, agroinfiltration of plants carrying the N resistance gene for expression of p50, an avirulence factor encoded by TMV, results in a rapid and severe HR, leading to death of infiltrated leaf areas within 36–48 h [43]. In a similar experimental system, BMB1 does not induce plant tissue death until 5 dpi. At earlier time points, the BMB1-induced response limits viral cell-to-cell transport in the infected tissue, most likely due to BMB1-induced callose deposition in the cell wall, limiting trafficking through the PD channels. In addition, the inhibitory effect of BMB1 on virus transport is found to be more pronounced at higher BMB1 expression levels. These observations suggest that BMB1 is involved in a compatible interaction with *N. benthamiana* that induces a defense response in a dose-dependent manner. Similarly, the PVX TGB1 protein serves as a factor of compatible interaction with *N. benthamiana* and can activate HR when the amount of TGB1 in plant cells reaches a threshold level [5]. Another parallel between BMB1 and PVX TGB1 in the induction of plant defense response is the upregulation of the expression of 9-LOX, a characteristic gene of the oxylipin biosynthesis pathway known to positively regulate programmed cell death upon viral infection [35]. The 9-LOX-activated pathway is involved in the mounting of both a local response to pathogens and systemic acquired resistance [44]. In particular, the products of the 9-LOX-catalyzed lipid oxidation can induce the synthesis of brassinosteroids, which in turn activate callose deposition in the cell wall [45]. This mechanism may account, at least in part, for the observed BMB1-induced callose deposition.

The BMB-induced plant defense response is found to be inhibited when BMB1 is coexpressed with BMB2, suggesting that regulation of the relative levels of BMB1 and BMB2 during virus infection can modulate both the virus movement and induced plant response. The suppression of the BMB1-induced response by BMB2 can only partially be attributed to the BMB2-mediated targeting of BMB1 to PAMBs, a possible mechanism of BMB1 sequestration and removal from the active pool. These data imply that there is another way in which BMB2 suppresses the BMB1-induced response, the mechanism of which remains elusive. In any case, the ability of BMB2 to suppress the plant defense is consistent with the previously proposed TMV cell-to-cell transport model, which suggests that viral infection activates a plant defense that leads to callose deposition in the cell wall, limiting virus spread, while the TMV MP is able to counteract this response by recruiting β-1,3-glucanases that hydrolyze callose [46]. Recently, this model has been supported by findings that double-stranded RNA (dsRNA), a viral replication intermediate, can serve as an inducer of pattern-triggered immunity manifested, besides activation of other pathways, by enhanced callose deposition, and that TMV MP expression correlates with the inhibition of callose deposition [47]. Further work is required to determine whether the BMB2-specific inhibition of the defense response induced by BMB1 is based on a mechanism similar to that of the TMP MP-specific inhibition of dsRNA-induced callose deposition.

Previously, BMB1 has been reported to localize to both the cytoplasm and nucleoplasm [26]. Here, treatment of BMB1-expressing cells with leptomycin B, an inhibitor of nuclear export of proteins containing NES recognized by importin 1/CRM1 [38], is found to result in nuclear accumulation of BMB1. This observation suggests that BMB1 is capable of both nuclear import and export and likely undergoes constant nuclear-cytoplasmic shuttling, resulting in a dynamic equilibrium of protein levels in the two compartments. In the nucleus, BMB1 is found in subnuclear structures identified as Cajal bodies. As found previously, deletion of 22 C-terminal amino acid residues of BMB1 results in increased localization to these structures [32], likely due to a shift in the balance between the nuclear and cytoplasmic localization, suggesting that the C-terminal region of BMB1 may contain a signal that influences nuclear export. Coexpression with mRFP-fused Fib2, the marker localized to the nucleolus and Cajal bodies, results in accumulation of BMB1 in the nucleolus in addition to Cajal bodies. Such a change in BMB1 localization can be speculated to result from the interaction of BMB1 with Fib2. As shown previously, BSMV TGB1 interacts with Fib2, and knockdown of the *fib2* gene results in inhibition of BSMV cell-to-cell transport [19], suggesting a functional significance of this interaction. Taking into account the distant evolutionary relationship of BMB1 and TGB1 proteins and the postulated similarity in the mechanisms of BMB- and TGB-mediated virus transport [27,28], the nuclear localization of BMB1 and its putative interaction with Fib2, similar to TGB1, may promote BMB1-dependent virus transport. This hypothesis is supported by the observation that BMB1 with an artificially added NLS, which is predominantly localized in the nucleus, mediates more efficient virus cell-to-cell movement than wt BMB1. BMB1 with NLS added to the N-terminus is found to induce a significantly weaker defense response compared to wt BMB1, inducing less callose deposition and, thus limiting virus spread to a lesser extent. However, quantification of infection foci size and callose deposition for NLS-BMB1 and controls shows that the more efficient virus transport observed in the case of NLS-BMB1 can only partially be attributed to the weaker defense response, suggesting that the nuclear localization of BMB1 *per se* is a factor in the more efficient virus transport. This conclusion is supported by experiments with the NLS-BMB1 mutant T71N, which has a point mutation in the conserved motif I of the BMB1 NTPase/helicase domain. When added to wt BMB1, this mutant, which is dysfunctional in virus cell-to-cell transport and induces a weak defense response, causes more efficient virus movement compared to wt BMB1. Thus, the BMB1 function that provides efficiency for virus cell-to-cell transport indeed requires protein localization to the nucleus and can be uncoupled from the enzymatic activities involved in the conventional steps of viral transport, such as formation of movement-competent complexes with genomic RNA and interaction with PD. We hypothesize that BMB1 may interact with nuclear protein(s) to activate a cell mechanism that upregulates trafficking through the PD channels. Alternatively, in the nucleus, BMB1 may act similarly to the GRV ORF3 protein that interacts with Fib2, resulting in the partial export of Fib2 to the cytoplasm, probably in the form of a complex with the ORF3 protein, and the formation of complexes of virus genomic RNA with Fib2 and the ORF3 proteins, representing a form of viral genome destined for systemic transport [21,22]. Similarly, BMB1 may be responsible for the formation of Fib2-containing complexes for virus cell-to-cell transport, as it has been proposed for BSMV TGB1, whose interaction with Fib2 contributes to the intercellular transport of virus infection [19]. This hypothesis fits the model suggesting that BMB1 undergoes nuclear-cytoplasmic trafficking, which may provide a mechanism for export of BMB1-Fib2 complexes from the nucleus.

In conclusion, this work demonstrates multiple levels of interactions between virus pathogens and their plant hosts during virus cell-to-cell transport. The HGSV BMB1 protein, in addition to its primary function in viral cell-to-cell transport, is shown to have two activities that modulate the efficiency of BMB-mediated transport. These activities have opposite effects, as the BMB1-induced defense response suppresses virus transport, whereas BMB1 targeting to the nucleus enhances virus transport, revealing a complex regulation of virus cell-to-cell spread that depends on BMB1 interactions with cell factors that may be unrelated to the virus movement function itself. An additional level of complexity is revealed by the ability of BMB2 to inhibit the BMB1-induced defense response. Analysis of the complex interactions during BMB-mediated virus cell-to-cell transport is the goal of future experimental work.

## 4. Materials and Methods

### 4.1. Molecular Cloning and Recombinant Constructs

The recombinant constructs for the transient expression of BMB1, BMB2, GFP-BMB1, GFP-BMB1d22, BMB2mutN, mRFP, PVX-POL-GFP, and mRFP-Fib2 were described previously [26,32,48,49].

All primers used for cloning are listed in Supplementary Table S1. Point mutations were introduced into the BMB1 construct by overlap PCR to obtain pLH-GFP-BMB1-mut1, pLH-GFP-BMB1-mut2, pLH-GFP-BMB1-mut3, and pLH-GFP-BMB1-mut4. In the first step, the PCR products were obtained by using the previously described pLH-GFP-BMB1 as a template with primer Left and one of the following primers, BMB1-mut1-M, BMB1-mut2-M, BMB1-mut3-M, or BMB1-mut4-M. Another set of PCR products was obtained with the same template and primers Right and one of the primers BMB1-mut1-P, BMB1-mut2-P, BMB1-mut3-P, or BMB1-mut4-P. In the second step, corresponding pairs of PCR products were fused together by amplification with primers Left and Right. The resulting DNA fragments for BMB1-mut1 and BMB1-mut2 were digested with *Xho*I*-Bgl*II and cloned into the similarly digested construct pLH-GFP-BMB1. The DNA products for BMB1-mut3 and BMB1-mut4 were digested with *Bgl*II*-Xba*I and cloned into the similarly digested construct pLH-GFP-BMB1.

To obtain TRV-BMB1, the coding sequence of BMB1 was amplified with primers BMB1-TRV-Mfe-P and BMB1-TRV-Xho-M using pLH-BMB1 [26] as a template. The PCR product was digested with *Mfe*I-*Xho*I and cloned in a TRV RNA2-based vector [33] digested with *Eco*RI-*Xho*I.

To obtain pLH-T71N-BMB1, overlap PCR was used. In the first step, a PCR product was obtained by using the previously described pLH-BMB1 construct as a template with primer Left and T71N-M, and another PCR product was obtained with primer Right and T71N-P. In a second step, two PCR products were fused together by amplification with the primers Left and Right; the resulting DNA product was digested with *Xho*I*-Xba*I and cloned into the similarly digested binary vector pLH* [50].

To obtain pLH-NLS-BMB1 and pLH-NLS-T71N-BMB1, the NLS-encoding fragment was obtained by the annealing and extension of primers NLS-M and NLS-P and digested with *Xho*I*-Sac*I, while the BMB1 or T71N-BMB1 coding sequences were amplified with primers BMB1-P-SacI and Right and digested with *Sac*I*-Xba*I. Both restriction products were cloned into the binary vector pLH* and digested with *Xho*I*-Xba*I. To obtain pLH-NES-BMB1, pLH-NES2-BMB1, and pLH-Flag-BMB1, the NES, NES2, and FLAG DNA fragments were obtained by the annealing and extension of pairs of specific primer pairs (Supplementary Table S1) and digested with *Xho*I*-Sac*I, while the BMB1 coding sequence was amplified with primers BMB1-P-SacI and Right and digested with *Sac*I*-Xba*I. Both restriction products were cloned into the binary vector pLH* and digested with *Xho*I*-Xba*I. To obtain the GFP-fused NES and NES2 constructs, the GFP coding sequence was cut out by *Xho*I*-Bam*HI digestion from the previously described GFP-BMB2 [26] and cloned into the similarly digested pLH-NLS-BMB1, pLH-NES-BMB1 or pLH-NES2-BMB1. To obtain pLH-GFP-Flag-BMB1, the GFP coding sequence was cut out by *NcoI* digestion from pLH-GFP-BMB1 and cloned into the similarly digested pLH-Flag-BMB1. All constructs were verified by sequencing.

### 4.2. Protein Sequence Analysis by Bioinformatics Tools

Predictions of the regions located on the surface of a protein molecule were made using the following web-based services: NetSurfP [51], PredictProtein [52], WESA [53], DeepREx-WS [54], and SVMTriP [55].

### 4.3. Plant Material

The *N. benthamiana* plants were grown and maintained in growth chambers under 16 h 24 °C day/8 h 20 °C night conditions and approximately 50% humidity. The 5 to 6-week-old plants were used for agrobacterial-mediated transient protein expression and movement complementation assays.

### 4.4. Plant Agroinfiltration

*Agrobacterium tumefaciens* (strain C58C1) cells were transformed with binary vectors using a standard freeze–thaw method. Prior to agroinfiltration, the bacterial cultures were grown and prepared as described previously [50]. Overnight cultures of agrobacteria were grown in the Luria–Bertani (LB) medium at 28 °C with selective antibiotics, 10 mm 2-(*N*-morpholino)ethanesulfonic acid (MES), pH 5.5, and 20 μm acetosyringone, pelleted, resuspended in the infiltration medium (10 mM MES, pH 5.5, 10 mM MgCl_2_, and 150 mM acetosyringone), and incubated at room temperature for 3–4 h. For infiltration, the *A. tumefaciens* suspensions were diluted to a final OD_600_ = 0.3. For the movement complementation tests, PVX-POL-GFP was infiltrated at OD_600_ = 0.00015 to obtain individually transformed plant cells. The agrobacterial cultures were infiltrated onto the abaxial surface of *Nicotiana benthamiana* leaves using a needleless syringe.

### 4.5. Aniline Blue Staining

To visualize the callose depositions, leaf sections were vacuum infiltrated with the aniline blue solution (0.1% aniline blue in 67 mM phosphate buffer, pH 8) and incubated in the dark at room temperature for 30 min before microscopy [56].

### 4.6. DAB Staining

On the third day, post infiltration, the leaves were vacuum infiltrated and incubated in 1 mg/mL of DAB (pH 3.8) for 6 h. After staining, the leaves were cleared in 96% ethanol to eliminate the background green color.

### 4.7. Leptomycin B Treatment

For the Leptomycin B treatment assay, at the end of the second day, post agrobacterial infiltration, the infiltrated leaves were infiltrated again with a 50 nM Leptomycin B solution. Confocal microscopy was carried out 10 h after the infiltration with Leptomycin B.

### 4.8. Confocal Microscopy and Virus Movement Visualization

Fluorescent proteins were visualized at 2–3 days after agroinfiltration in epidermal cells. Excised leaf sections were vacuum infiltrated with water and observed using a confocal laser scanning microscope Nikon C2plus (Tokyo, Japan) equipped with a ×60 (1.2 NA) water immersion objective. The excitation wavelengths were 488 nm for GFP, 543 nm for RFP, and 405 nm for aniline blue. The images were acquired at 495–545 nm for GFP, at 580–640 nm for RFP, and at 416–490 nm for aniline blue and processed using Nikon NIS Elements software (v. 4.20). In the complementation assays, the cell-to-cell movement of PVX-POL-GFP was observed using a confocal laser scanning microscope Nikon C2plus equipped with a ×10 objective, and the foci sizes were measured using Nikon C2plus software (v. 5.41.01). Quantification of callose at PD was obtained using the CalloseQuant plugin for ImageJ (v. 1.54) [57]. Specifically, regions of interest (ROI) were selected automatically using Method A with the following parameters: the peak prominence value was set to 100, and the measurement radius was set to 4. Further, the selected ROIs were manually checked to ensure that only PD-associated aniline blue-stained foci were included in the analysis. For the statistical analyses of data, a nonpaired two-tailed Student’s t-test was applied. In the virus movement complementation assays, agroinfiltrated plant leaves were observed under long-wave UV light (365 nm) using a Black-Ray B-100AP lamp (UVP, Cambridge, UK).

### 4.9. Quantitative PCR

Leaf sections were frozen in liquid nitrogen and ground to fine powder. Total RNA was extracted using the ExtractRNA reagent (Evrogen, Moscow, Russia) according to the manufacturer’s instructions. To prevent contamination of RNA samples with plant genomic DNA, the samples were thoroughly treated with RNase-free DNAseI (ThermoFisher Scientific, Waltham, MA, USA). The total RNA samples were reverse-transcribed into cDNA with the oligo(dT) primer using a Revertaid reverse transcriptase (ThermoFisher Scientific) according to the manufacturer’s instructions. The obtained cDNAs were used as a template for real-time PCR reactions conducted with the qPCRMix M-440 (Synthol, Moscow, Russia) and 9-LOX mRNA-specific primers or primers specific for F-box mRNA, which was chosen as the reference gene (Supplementary Table S1). The real-time PCR assays were performed in the CFX Connect Real-Time PCR System (Bio-Rad, Hercules, CA, USA). The *C*_t_ value for 9-LOX mRNA was normalized to the reference gene mRNA. For the statistical analyses of data, a nonpaired two-tailed Student’s *t*-test was applied.

## Data Availability

Data are contained within the article and Supplementary Materials.

## Supplementary Materials

The following supporting information can be downloaded at: https://www.mdpi.com/article/10.3390/plants13182550/s1, Figure S1, BMB1 induces plant defense response when expressed from the TRV vector; Figure S2: Subcellular localization of GFP-fused BMB1 mutants with point mutations in predicted protein surface-located regions; Figure S3: Schematic representation of BMB1 derivatives carrying artificially added nuclear trafficking signals; Figure S4: Induction of plant defense response by BMB1-T71N and NLS-BMB1-T71N; Table S1: Primers used in this study.
