# Supplementary material for: Defense Responses Induced by Viral Movement Protein and Its Nuclear Localization Modulate Virus Cell-to-Cell Transport
Source: Plants (Basel). 2024 Sep 11;13(18):2550. doi: 10.3390/plants13182550 (PMC11435296; doi:10.3390/plants13182550)
Supplement: Supplementary file 1 [file plants-13-02550-s001.zip › plants-3166806-supplementary.pdf]

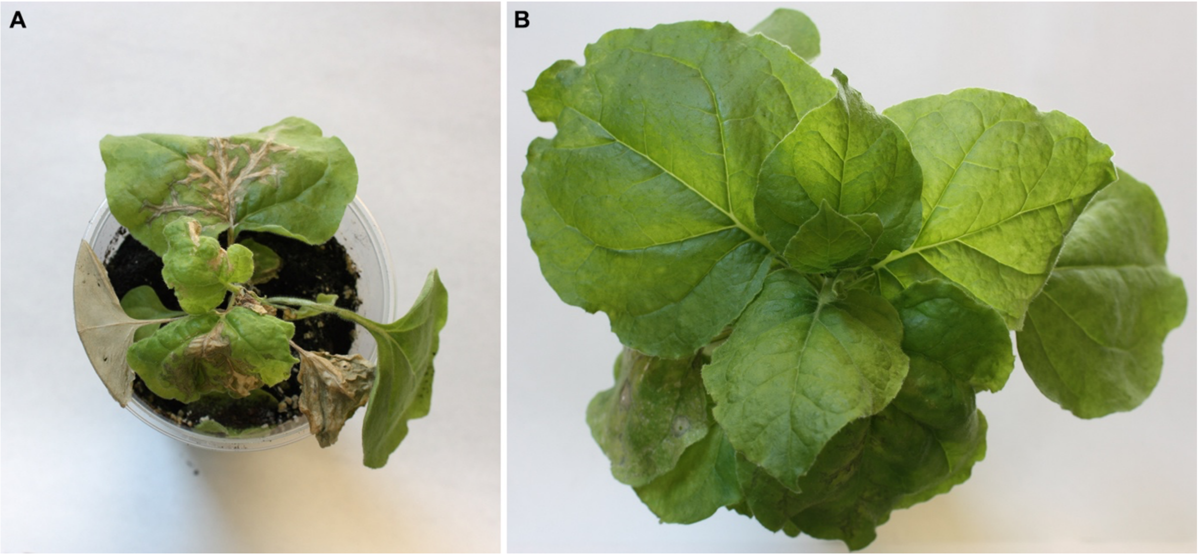

Supplementary Figure S1. BMB1 induces plant defense response when expressed from TRV vector. Images show *N. benthamiana* plants simultaneously infected with either TRV-BMB1 (A) or empty TRV vector (B). Images were taken at 11 dpi.

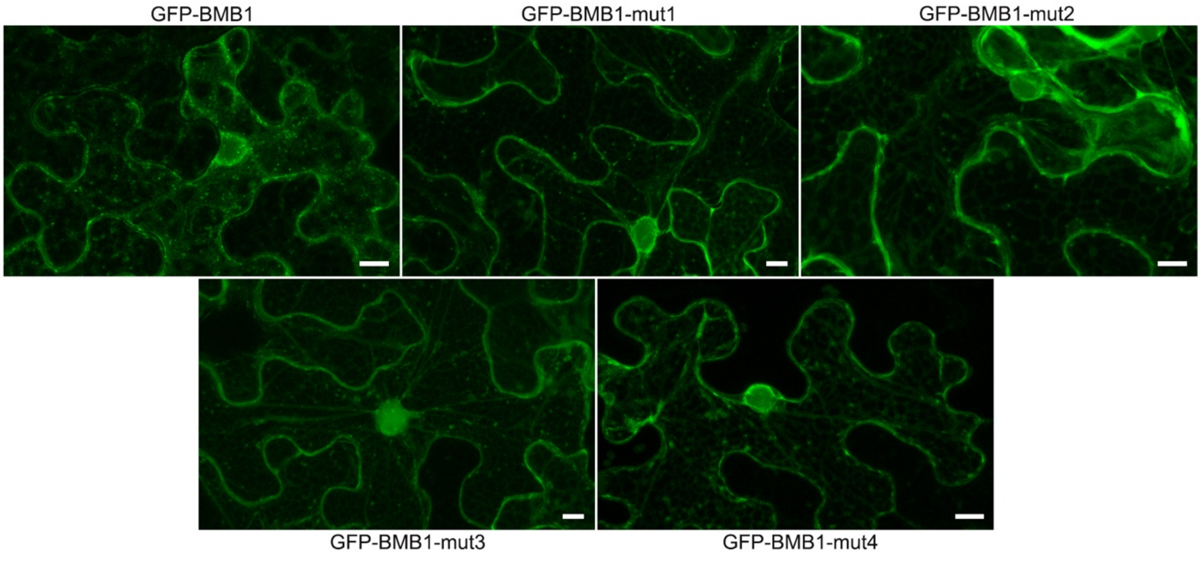

Supplementary Figure S2. Subcellular localization of GFP-fused BMB1 mutants with point mutations in predicted protein surface-located regions.

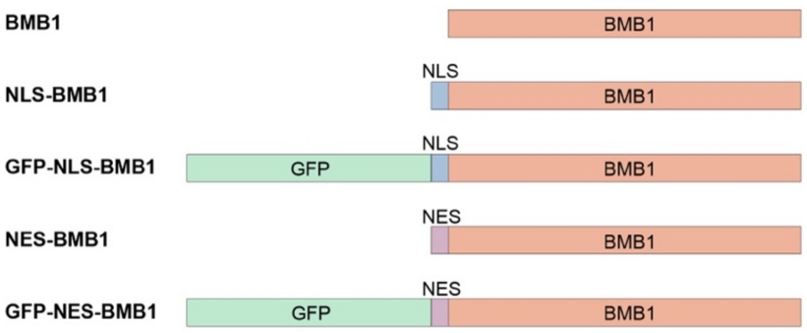

Supplementary Figure S3. Schematic representation of BMB1 derivatives carrying artificially added nuclear trafficking signals. Rectangles represent proteins. NLS shows the position of the SV40 T-antigen NLS (PKKKRKVEDP). NES depict the position of both the cAMP-dependent protein kinase inhibitor protein NES (ELALKLAGLDIN) and the HIV-1 Rev NES (LQLPPLERLTL).

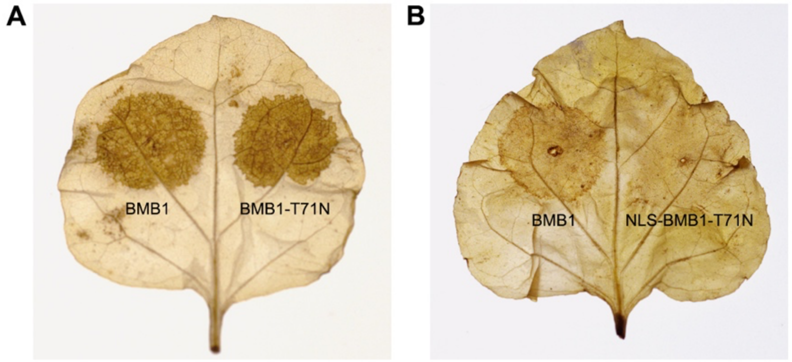

Supplementary Figure S4. Induction of plant defense response by BMB1-T71N (A) and NLS-BMB1-T71N (B). Leaves were agroinfiltrated for expression of BMB1, BMB1-T71N, and NLS-BMB1-T71N and stained with DAB at 3 dpi.

Supplementary Table S1. Primers used in this study.

Amplification of inserts on pLH*-based constructs	
Left	TATCCTTCGCAAGACCCTTCCTCT
Right	CCTTATCTGGGAACTACTCACACATT
GFP-BMB1-mutants cloning	
BMB1-mut1-M	CTATAACCACCACTCCTATACTCATCC
BMB1-mut1-P	GAGTATAGGAGTGGTGGTTATAGGAGT
BMB1-mut2-M	GCAGAGCTAACAGCTGGTAGTGC
BMB1-mut2-P	CTACCAGCTGTTAGCTCTGCTGA
BMB1-mut3-M	CCAGAGAGGATCCGGAAGCACCG
BMB1-mut3-P	GCTTCCGGATCCTCTCTGGAGTCT
BMB1-mut4-M	GCCCGGAGACGTGCCCTGTAAAC
BMB1-mut4-P	AGGGCACGTCTCCGGGCTGGTAG
T71N-BMB1 cloning	
T71N-M	CATACACAAACTTGTTCTTACCACCAC
T71N-P	GTGGTGGTAAGAACAAGTTTGTGTATG
NLS-BMB1 and NLS-T71N-BMB1 cloning	
NLS-M	CAGAGCTCCCAGGGTCTTCTACCTTTCTTTTCTTCTTTGGG
NLS-P	GGCTCGAGACCATGGGATCCCCAAAGAAGAAAAGAAAGGTA
NES-BMB1 cloning	
NES-M	CAGAGCTCCCATTAATATCCAATCCAGCAAGCTTCAATGCAAG
NES-P	GGCTCGAGACCATGGGATCCGAACTTGCATTGAAGCTTGCTGG
NES2-BMB1 cloning	
NES2-M	CAGAGCTCCCCAAAGTAAGTCTTTCCAATGGAGGCAATTGAA
NES2-P	GGCTCGAGACCATGGGATCCCTTCAATTGCCTCCATTGGAAA
Flag-BMB1 cloning	
FLAG-Sac-M	CAGAGCTCTTACCCTTGTCATCGTCATCCTTGTAATCAGAG
FLAG-Xho-P	GGCTCGAGACCATGGGAAGCTCTGATTACAAGGATGACGAT
Fusion of NLS, NES, NES2, and FLAG to BMB1	
BMB1-P-SacI	GGGAGCTCTGTGGAGAGTTTTAATTATGTGACTTC
BMB1-TRV cloning	
BMB1-TRV-Mfe-P	GCGCAATTGACCATGGAGAGTTTTAATTATGTGACTTC
BMB1-TRV-Xho-M	GCCTCGAGTTAAAGTACGTAAAAATCACCTCTCA
Detection of 9-Lox mRNA	
Nb9LOX-det-R	TCTTGAGCTCTTCATACGCGGG
Nb9LOX-det-F	GATAAGAAAGACGAGCCTTGGTGG
Detection of F-box mRNA	
F-Box-F	GGCACTCACAAACGTCTATTTC
F-Box-R	ACCTGGGAGGCATCCTGCTTAT
